# Can digital prompting and the engagement of the husband influence the satisfaction of disadvantaged women with their reproductive health journey? A cross-sectional study from Lebanon

**DOI:** 10.1177/20552076251406650

**Published:** 2025-12-17

**Authors:** Shadi Saleh, Nadine Sabra, Nour El Arnaout, Asmaa El Dakdouki, Khaled El Iskandarani, Zahraa Chamseddine, Mohamad Alameddine

**Affiliations:** 1 Global Health Institute, 11238 American University of Beirut, Beirut, Lebanon; 2 Department of Health Management and Policy, Faculty of Health Sciences at the American University of Beirut (AUB), Beirut, Lebanon; 3 College of Health Sciences, 59105 University of Sharjah, Sharjah, United Arab Emirates

**Keywords:** Maternal health, gamification, artificial intelligence, mHealth, disadvantaged women, antenatal care (ANC), healthcare provider, digital prompting, reproductive health

## Abstract

**Objective:**

Maternal health in Lebanon is severely impacted by the country's ongoing socioeconomic crisis, disproportionately affectivng disadvantaged Lebanese and refugee women due to limited healthcare access. Digital prompting interventions have improved antenatal and postnatal care utilization, particularly when the husband of the pregnant woman is also engaged. This study aims to assess the influence of digital prompting and husband engagement on the satisfaction of disadvantaged pregnant women in Lebanon with their reproductive health journeys, using the artificial intelligence (AI)-based gamified mHealth intervention titled “Gamification and Artificial Intelligence and mHealth Network for Maternal Health Improvement” (GAIN MHI).

**Methods:**

This study was conducted across seven primary healthcare centers in Lebanon, targeting pregnant women up to 16 weeks of gestation with mobile phone access. The intervention included digital messages for both pregnant women and their husbands, alongside the GAIN MHI App for healthcare providers. Over 11 months, data was collected to assess maternal satisfaction, antenatal care (ANC) attendance, and the role of husband engagement in supporting maternal wellbeing.

**Results:**

A total of 1028 pregnant women participated. Husband involvement significantly improved support for ANC visit, reminder's frequency, and psychological support. Women receiving mobile health support were more likely to report better physical health (odds ratio (OR) = 2.16; p = 0.03) and mental health (OR = 2.12; p = 0.03). Increased ANC visits were associated with higher likelihood of satisfaction with baby health (OR = 1.35; p = 0.05) and with service quality (OR = 2.68; p = 0.01). Husband support for ANC visits improved satisfaction both predelivery (OR = 2.15; p < 0.01) and postdelivery (OR = 2.05; p < 0.01). The combined effect of all support factors significantly boosted satisfaction with self-care predelivery (OR = 2.07; p < 0.01) and postdelivery (OR = 3.82; p < 0.01).

**Conclusion:**

The findings emphasize the importance of hybrid digital health models integrating mobile-based education, spousal support, and healthcare provider engagement to enhance maternal satisfaction and health outcomes. Future programs should adopt this approach to ensure comprehensive maternal care.

## Introduction

### The state of maternal health in Lebanon

Maternal mortality persists as a critical global health challenge, with pronounced disparities observed across socioeconomic strata.^1,2^ Women in low- and lower-middle-income countries (LLMICs) endure a disproportionate burden of pregnancy-related deaths, primarily attributable to preventable complications during gestation, childbirth, and the postpartum period.^3,4^ This disparity intensifies in humanitarian crises, since conflict-affected and displaced populations are most vulnerable to adverse health outcomes.^4,5^ Lebanon exemplifies this humanitarian crisis compounded by a socioeconomic collapse that has severely compromised access to healthcare for both disadvantaged Lebanese and refugee populations.^6^ An alarmingly high rate of obstetric complications was documented among Syrian refugee women in Lebanon. This was directly correlated with insufficient utilization of essential maternal health services.^7–9^ Such complications are exacerbated by persisting critical gaps in access to skilled birth attendants, postnatal support, and suboptimal antenatal care visits (ANC).^10^ In particular, ANC visits are adversely affected by poor financial status, limited knowledge on the importance of seeking healthcare,^11,12^ and constrained autonomous decision making due to gender inequality.^13^ Given that women's health was identified as one of the main health needs among refugees in Lebanon,^14^ promoting ANC^15^ and comprehensive health promotion throughout pregnancy, delivery, and the postpartum period is crucial.^16^ In this regard, digital health interventions hold promise for enhancing access to essential maternal health services.^17,18^

### Digital prompting for improved maternal health outcomes

In LMICs, ubiquitous technologies such as mobile phone networks and devices are being leveraged to bridge gaps in healthcare access, quality, and efficiency.^19,20^ Digital health interventions, including mobile health (mHealth), have demonstrated significant potential in addressing maternal and child health challenges.^17,18^ Specifically, digital prompting—the use of digital technologies to send periodic messages to encourage health behaviors—has empowered pregnant women and mothers with educational content and appointment reminders, driving increased ANC and postnatal care (PNC) visits and improved healthcare seeking behaviors.^17,18^^21–25^ For instance, Promoting Mothers through Pregnancy and Postpartum (PROMPTS), an artificial intelligence (AI)-enabled short message services (SMS) platform in Kenya, delivers stage-based guidance and connects mothers to clinical support, significantly improving antenatal and PNC adherence in informal settlements.^26^ Beyond PROMPTS, broader evidence highlights that SMS-based digital prompting not only increased ANC and PNC attendance^27–29^ but also reduced perinatal mortality,^30,31^ and enhanced women's healthcare decision making and knowledge.^32^ However, maternal health choices are often mediated by spouses, who may influence healthcare-seeking or resource allocation.^33^ While digital prompting for pregnant women has proven effective, its impact could be amplified by engaging women's social networks, particularly their spouses.

### Engaging spouses in maternal health through digital prompting

Maternal health outcomes are influenced by gender dynamics, as limited decision-making autonomy and restricted financial access worsen maternal health outcomes in many LMICs.^34–42^ For instance, it is common for women to require their husband's permission to leave home or to be accompanied by him when traveling.^38,39,41,42^ This practice not only limits their ability to seek care promptly but also reinforces dependency on their husbands for financial support, covering costs such as transportation to healthcare facilities.^35,36^^38–42^ Additionally, most refugee women lack independent income, which compounds these barriers, leaving them more vulnerable when trying to access necessary maternal health services.^34–36^^,38–42^ Husbands often hold primary decision-making power in healthcare matters, where their involvement in maternal health can improve access to ANC and PNC, increase emotional support, and foster shared decision making, enhancing maternal health outcomes.^43–50^ For these reasons, the engagement of the husband in the empowerment of women in seeking maternal healthcare is crucial to mitigate preventable maternal morbidity and mortality.^50^ One promising approach to achieving this is through digital prompting interventions, by delivering targeted educational messages for both pregnant women and their spouses.^51,52^ These interventions may improve knowledge and understanding of maternal health, while also promoting greater spousal support.^53,54^ For example, in Afghanistan, the Mobile Alliance for Maternal Action “MAMA” program implemented a digital prompting intervention targeting both pregnant women and their husbands.^54^ This program conveys educational messages on maternal, newborn, and child health (MNCH), boosting healthy behaviors and care seeking for pregnant women and their infants up to 1 year old.^54,55^ Assessment of the implementation of this program showed an increase in joint decision making on MNCH between partners.^54^ In addition, their knowledge concerning pregnancy warning signs, breastfeeding, and lifesaving MNCH issues were ameliorated.^54^ Even when husbands were engaged independently of their wives, health-seeking behaviors improved. For instance, in India, biweekly messages sent to husbands increased the likelihood of their wives attending an antenatal checkup in the third trimester and receiving a postnatal checkup within 7 days of delivery.^53^

### State of digital prompting for maternal health in Lebanon

Digital prompting interventions have been increasingly adopted in Lebanon, reflecting a broader push toward leveraging technology for improving healthcare service delivery and health outcomes.^56^ Educational text messages and appointment reminders were implemented to effectively manage noncommunicable diseases (NCDs) like hypertension and diabetes in underserved communities.^57^ An example of a digital prompting intervention for maternal health in Lebanon is the electronic mother and child health (e-MCH) application, developed by United Nations Relief and Works Agency (UNRWA), which offers pregnant women and mothers in refugee settings with appointment reminders, helping them to maintain uninterrupted access to essential healthcare services.^58^ However, digital prompting initiatives that specifically address maternal health are scarce, leaving expectant mothers with limited tailored digital support during critical periods of their reproductive health journey.^56^ Additionally, there are currently no digital prompting interventions that engage spouses while targeting maternal health.^45,59^ This gap underscores the urgent need for an innovative digital prompting solution that not only focuses on maternal care but also actively involves spouses, to better understand whether spousal engagement in digital health interventions plays a role in influencing health seeking behavior of expectant mothers in Lebanon.

### Aim of the study

The aim of this study is to evaluate the satisfaction of disadvantaged pregnant women in Lebanon with their reproductive health experiences following exposure to a digital intervention using an AI-based, gamified mHealth platform titled “Gamification and Artificial Intelligence and mHealth Network for Maternal Health Improvement.” Specifically, it aims to investigate the impact of digital prompting and husband engagement on satisfaction levels.

## Methodology

The GAIN MHI trial is a community-based interventional study with historical controls conducted across 19 primary healthcare centers (PHCs) over a 16-month period (September 2021–December 2022) in Lebanon. It evaluated the impact of a multimodal maternal health intervention, comprising gestational age–tailored mobile messages (text and voice) for the pregnant women and their spouses, and a gamified, AI-driven professional development app for healthcare providers, on maternal and neonatal outcomes. Nineteen PHCs serving disadvantaged Lebanese and refugee populations were enrolled, where PHCs were first matched by intervention sites across the UNRWA and Lebanese Ministry of Public Health (MOPH) networks and then randomized within each matched pair to intervention or control, ensuring balance in service context. As a result, 9 centers were assigned to the intervention arm and 10 to the control arm.

Our study is a cross-sectional analytical study, nested within the intervention arm of the parent trial. It was conducted postintervention across the two arms: the first consisting of pregnant women that received the intervention, and the second group consisting of pregnant women that received the intervention along with their spouses.

### Study design and settings

The study was conducted across eight PHCs located in various governorates in Lebanon, incorporating the South, North, Beqaa, and Beirut regions. Of the eight PHCs, four were administered by the UNRWA, which primarily serves Palestinian refugees, while the remaining four were operated by the MOPH, serving disadvantaged Lebanese and Syrian refugees. However, due to logistical challenges, one PHC was excluded, reducing the total number of PHCs to seven. The intervention extended over a period of 16 months (from September 2021 until December 2022).

### Study population

The study targeted pregnant women attending one of seven participating PHCs in Lebanon between September 2021 and December 2022. Inclusion criteria for this cross-sectional analytical study were pregnant women who were eligible, participated, and completed the GAIN MHI intervention. The inclusion criteria for the initial parent community interventional trial were pregnant women with a gestational age of up to 16 weeks, who owned or had reliable access to a mobile phone, and were seeking care at one of the selected PHCs during the recruitment period. Women beyond 16 weeks’ gestation, those without reliable phone access, or those who declined to consent were excluded from the parent community interventional trial. At four of the PHCs, male partners were also invited to participate in the intervention, provided they gave written consent. However, the primary study population consisted of pregnant women.

### The mHealth intervention

In the formative phase, a total of 64 educational messages for each pregnancy week and up to 6 months after delivery were co-created with a number of refugees and disadvantaged Lebanese women and their spouses through focus group discussions with the target population. These messages were developed in Arabic using terms that are understandable by, and suit the context of, the targeted population. Subsequently, a validation and pilot process for messages directed at pregnant women were executed with another group of refugees and disadvantaged Lebanese women who are currently pregnant or have been pregnant once before. Similarly, messages intended for husbands were validated and piloted among a group of husbands of refugee and disadvantaged Lebanese women. Messages for both were then adjusted according to participants’ feedback. Additionally, participants were asked about their preferred message format (text or voice), where they revealed a preference for receiving both text and voice messages. Consequently, messages were delivered in both formats to accommodate their preferences.

#### Intervention design

The intervention was structured into two arms: one focusing solely on pregnant women and the other incorporating both pregnant women and their husbands. Arm assignment was determined at the PHC level by the matched-pair randomization, where spouses did not self-select. A key distinguishing component of this intervention is the integration of digital prompting, including text and voice messages delivered in Arabic, to the phone numbers provided by participants. Although not the primary focus here, healthcare providers were targeted through an AI-driven, gamification-based mobile application “GAIN MHI App” designed to enhance their knowledge and improve maternal health outcomes.

#### Intervention targeting pregnant women

Messages for pregnant women were included in both arms of the intervention, and were identical in content (Arm 1 targeted pregnant women only, while Arm 2 included both pregnant women and their spouses). These messages included the following components:

- **Weekly gestational age-specific educational messages**: Delivered pregnancy education, including several topics such as nutritional recommendations, supplement intake, and pregnancy danger signs.
- **Postpartum education messages for a duration of 6 months after delivery**: To promote postpartum visits, breastfeeding, contraception, maternal mental health, and infant vaccination.
- **Personalized reminder messages**: To notify women of their upcoming ANC visits, required vaccinations, tests, and medical examinations.

#### Intervention targeting husbands of pregnant women

Weekly mobile-based informative and reminder messages were delivered to spouses similar to those received by their wives. Additionally, weekly informative messages regarding the emotional and psychological paternal support during pregnancy were also conveyed to them. The aim was to explore how gender dynamics can improve the effectiveness of mHealth interventions targeting pregnant women.

#### Intervention targeting healthcare providers

The GAIN MHI App leverages gamification and AI to enhance healthcare providers’ professional development. It features a trivia-based format with monthly recognition (most improved player and most valuable player) and monetary incentives to encourage engagement. AI-driven algorithms identify knowledge gaps from incorrect responses, enabling personalized learning across five maternal health domains: Prevention, Diagnosis, Management, Miscellaneous, and COVID-19. Users interact with a spin-wheel question selection, receive explanations for answers, and access a knowledge resource center. Automated WhatsApp messages provide feedback on scores, attempts, performance, and rewards. Available in English and Arabic, the app offers an innovative approach to improving maternal healthcare education.

Although the intervention included the professional development of HCPs, this aspect is not the primary focus of this study and is explored in more detail in a separate paper.^60^

### Data collection

The data collection process was extended over 11 months (from October 2022 until August 2023). Since participants were recruited to the study at different gestational ages, some had not delivered yet at the time of data collection and were still receiving ANC messages, while others had received the full set of messages, including PNC messages. Only those who completed the full intervention messages were eligible for data collection. Of the 1565 pregnant women enrolled, 1028 were eligible for the study which was conducted via phone calls. Although 1028 women completed the full intervention, the satisfaction questions were applicable only to the 683 women who had at least one prior pregnancy. The survey questions were administered in a manner that concealed the main hypothesis from women. In other words, participants were blinded to the main hypothesis of the survey aiming at avoiding misclassification bias. The survey assessed PHC experience, husband frequency of support, and satisfaction with the pregnancy and postpartum journey and with the role of intervention in contributing to the satisfaction. Assessment factors of satisfaction were evaluated based on the satisfaction of a pregnant woman with her mental health, physical health, baby health, self- and fetus care predelivery, self-care postdelivery, and quality services with respect to previous pregnancy experience. The assessment factors of satisfaction were categorized into two levels: improvement versus no difference or worse.

### Statistical analysis

Statistical analysis was conducted using Stata Software (SE version) for Windows. The demographic characteristics of the participants, ANC utilization, the role of the husband, and satisfaction assessment factors were summarized and presented using descriptive statistics. Categorical variables were expressed as counts and percentages, while continuous variables were summarized as means with standard deviations to provide an overview of the data distribution. To assess the association between husband enrollment in the intervention and the husband's role in pregnancy-related activities, a chi-square test was employed. This test helped to evaluate if the husband's involvement (e.g. support for attending ANC visits, reminders, and psychological support) differed significantly between those who were enrolled in the intervention and those who were not.

Simple logistic regression models were used to quantify the influence of key variables—such as PHC visits, husband enrollment, husband support for ANC visits, frequency of support, psychological support, and type of support—on each of the satisfaction assessment factors (such as satisfaction with physical health, mental health, self-care, and service quality). These models provided insight into the odds of improvement in satisfaction based on each of the predictor variables, measured independently for each factor. A p-value of <0.05 was considered statistically significant, indicating that the relationships observed between the variables and satisfaction outcomes were unlikely to have occurred by chance. This approach allowed for a thorough exploration of the factors that could influence pregnant women's satisfaction with their care, providing valuable insights into the roles of both healthcare services and spousal involvement.

### Ethical considerations

Ethical approval was sought and obtained from the Institutional Review Board at the American University of Beirut (SBS-2020-0317) and the respective ethical committees at MOPH and UNRWA. Eligible pregnant women who agreed to be part of the study signed a consent form. Additionally, at four of the selected PHCs, the husbands of recruited women were also invited to participate and were asked to provide a written consent to enroll in the study.

## Results

Table 1 presents the baseline demographic characteristics of 1028 pregnant women. The mean age was 28.3 ± 5.9 years and all patients in this study were females. The majority of the participants were Palestinian, while 274 (17.5%) were Syrian and 189 (12.1%) were Lebanese. The mean number of pregnancies (Gravida) among the participants was 3 ± 1.7. In terms of residence, 272 participants lived in urban areas (26.5%), while 756 (73.5%) resided in rural areas. Among the study population, 1028 participants responded completely to the questions about ANC utilization and satisfaction. Of these, 1018 women had attended 4 or more ANC visits. Regarding the encouragement for ANC visits, 306 women (29.8%) reported that no one specifically encouraged them to attend ANC visits. Furthermore, 5.3% identified HCP and 5.6% cited digital prompts as sources of encouragement. Additionally, 142 out of 1028 women (13.8%) indicated that their husbands encouraged them, while 468 (45.5%) believed that a collective approach, involving multiple factors, played a role in encouraging their attendance. In addition, 683 (66.4%) reported that the current pregnancy is not their first, and 40.34% of those reported an increase in PHC visits compared to previous pregnancies. Table 1 summarizes the demographics and ANC services.

**Table 1.** Demographics and antenatal care utilization patterns among pregnant women (n = 1028).

Variables		
Age (mean, SD)	28.3 (5.9)	
Gravida (mean, SD)	3.0 (1.7)	
Residence area (n, %)		
*Urban*	272	26.5
*Rural*	756	73.5
ANC visits (n, %)		
* Less than 4*	10	0.10
* 4 and more*	1018	99.0
Main encouragement for ANC visits (n, %)		
* No one*	306	29.8
* Healthcare worker/provider (HCP)*	54	5.3
* Mobile digital prompt*	58	5.6
* Women's husband*	142	13.8
* All factors combined*	468	45.5
First pregnancy (n, %)		
*No*	345	33.6
*Yes*	683	66.4
Increase in PHC visits compared to previous pregnancies (n, %)		
*No*	408	59.7
*Yes*	275	40.3

PHC:primary healthcare center.

### Husband role in the intervention

Table 2 outlines the various ways in which husbands were involved in their wives’ pregnancies as perceived by the pregnant women, including their support for their wives for attending ANC visits, the frequency of reminders for these visits, and the psychological support. Table 2 also explores the relationship between husband enrollment in the intervention and the level of involvement in the women's pregnancy journey, assessing whether this enrollment yielded significantly better involvement. These results highlight the positive impact of husband enrollment in the intervention, with significant improvements in support for attending ANC visits, the frequency of reminders, and psychological support.

**Table 2.** Husband involvement in pregnancy.

	All		Husband enrollment		
Variables	n	(%)	No n (%)	Yes n (%)	Significance
Husband enrollment in the intervention					
*No*	419	40.4			
*Yes*	607	59.6			
Husband support for Attending ANC visits					
*No*	107	11.3	57 (13.6)	50 (8.2)	.005*
*Yes*	918	88.6	361(86.4)	557(91.8)	
Frequency of husband's Reminders for ANC visits					
*Periodic or null reminder*	115	11.2	62 (14.8)	53 (8.7)	.002*
*Continuous reminder*	911	88.8	357(85.2)	554(91.3)	
Husband psychological Support					
*No*	109	10.6	55 (12.7)	54 (8.9)	.033*
*Yes*	919	89.4	366(87.3)	553(91.1)	

*Note:* Some numbers under some categories may not add up to the total because of missing values.

* Significant at 0.05.

**Figure 1.**
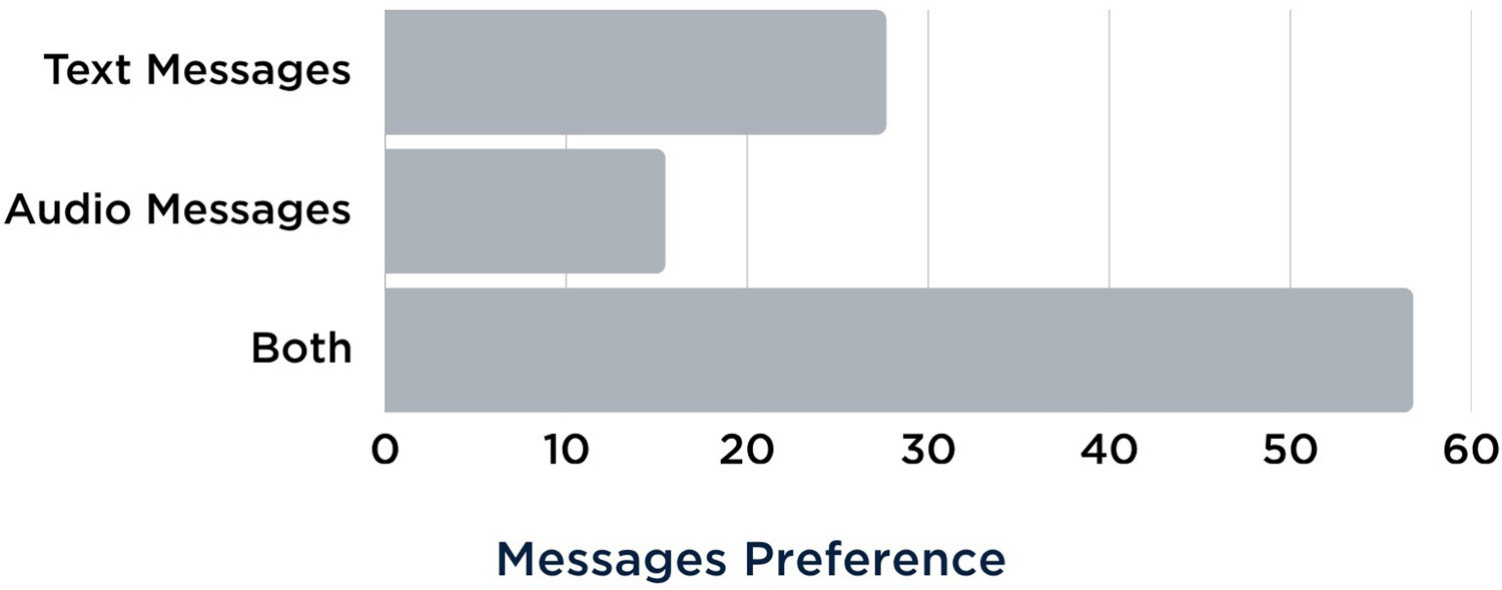


Table 3 illustrates participants’ perceptions of the messaging intervention. And 83%(n = 853) found the message frequency optimal. In terms of utility, 99.6% (n = 1024) deemed the content “very useful,” and all respondents found messages easy to read. Preferences spanned text (27%), audio (15%), or both formats (58%), indicating that multimodal delivery supports broad acceptability and illustrates the perceptions of pregnant women regarding their digital experience (Figure 1). All participants in the satisfaction survey found the messages easy to read.

**Table 3.** Table showing perceptions of pregnant women concerning intervention's messages.

Variables	n	(%)
Frequency of messages sent		
Optimal for knowledge Advancement	853	83.0
Ease of reading message		
Yes	1028	100.0
Utility of the message		
Very useful	1024	99.6
Not very useful	4	0.4
Message preference		
WhatsApp audio message	159	15.5
WhatsApp text message	284	27.6
Both	585	56.9

### Assessment of satisfaction factors

Table 4 shows the assessment factors selected to evaluate the satisfaction of pregnant women with the intervention compared to their previous pregnancy experiences with a sample size of 683 participants. For satisfaction with physical health, 65.7% of women reported improvement, while 34.3% noted no difference or worse. In terms of mental health, 67.2% of women felt improvement, whereas 32.8% did not experience any change or reported worsening. Satisfaction with self- and fetus care during pregnancy showed a similar trend, with 67.8% of women reporting improvement and 32.2% reporting no difference or worse. However, for satisfaction with the baby's health postdelivery, 45.4% of women saw improvement, while a higher percentage, 54.6%, indicated no difference or worse. Regarding self-care postpregnancy, 66.9% of women experienced improvement, while 33.1% reported no change or worsening. Finally, satisfaction with the quality of PHC services showed that 57.8% of women were satisfied with an improvement in service quality, while 42.2% noted no difference or a decline in quality.

**Table 4.** Assessment factors for the satisfaction of pregnant woman with current pregnancy compared to previous pregnancies (n = 683).

	Variables	n	(%)
Assessment factor 1	**Satisfaction with physical health**		
	** ** *Improvement^a^*	449	65.7
	** ** *No difference or worse*	234	34.3
Assessment factor 2	**Satisfaction with mental health**		
	*Improvement^a^*	459	67.2
	*No difference or worse*	224	32.8
Assessment factor 3	**Satisfaction with self- and fetus care during pregnancy**		
	*Improvement^a^*	463	67.8
	*No difference or worse*	220	32.2
Assessment factor 4	**Satisfaction with baby's health postdelivery**		
	*Improvement*	310	45.4
	*No difference or worse*	373	54.6
Assessment factor 5	**Satisfaction with self-care postpregnancy**		
	*Improvement^a^*	457	66.9
	*No difference or worse*	226	33.1
Assessment factor 6	**Satisfaction with quality of PHC services provided**		
	*Improvement*	395	57.8
	*No difference or worse^a^*	288	42.2

^a^The satisfaction assessment factor was defined as experiencing a better outcome in each indicator during the current pregnancy compared to the previous one.

PHC: primary healthcare center.

 Table 5 presents the factors associated with an increased likelihood of satisfaction among pregnant women, highlighting the odds ratios (OR) and their corresponding 95% confidence intervals (CI) for several influencing variables, including PHC visits, husband enrollment and support, frequency of support, psychological support, and types of support. In terms of satisfaction with physical and mental health, the type of support received was the primary factor associated with improvement. Mobile health support notably doubled the odds of improvement in physical health (OR = 2.16; CI = [1.09, 4.31]; p = 0.03) and mental health (OR = 2.12; CI = [1.06,4.23]; p = 0.03). Additionally, the collective impact of all factors showed significant improvement in both physical (OR = 2.15; CI = [1.47, 3.12]; p < 0.01) and mental health (OR = 2.34; CI = [1.6, 3.43]; p < 0.01).

**Table 5.** Odds ratio assessing the variables influencing assessment of satisfaction factors (improvement versus no difference or worse as reference).

	Assessment factor 1		Assessment factor 2		Assessment factor 3		Assessment factor 4		Assessment factor 5		Assessment factor 6	
Associated variables *(level of interest vs ref.)*	Satisfaction with physical health		Satisfaction with mental health		Satisfaction with self- & fetus care		Satisfaction with baby health		Satisfaction with self-care postdelivery		Satisfaction with service quality	
	OR (95% CI)	Sig.	OR (95% CI)	Sig.	OR (95% CI)	Sig.	OR (95% CI)	Sig.	OR (95% CI)	Sig.	OR (95% CI)	Sig.
PHC visits												
*Increase, vs no*	0.98 (0.71,1.35)	.91	0.93 (0.67,1.28)	.64	1.64 (1.15,2.25)	.01*	1.35 (1.00,1.38)	.05*	1.66 (1.19,2.32)	<.01*	2.68 (1.93,3.71)	.01*
Husband enrollment												
*Yes, vs No*	1.11 (0.81,1.53)	.52	1.03 (0.74,1.42)	.86	1.2 (0.87,1.66)	.27	1.02 (0.75,1.30)	.89	1.25 (0.91,1.73)	.18	1.26 (0.92,1.71)	.15
Husband support of ANC visits												
*Yes, vs No*	1.18 (0.74,1.87)	.49	1.27 (0.80,2.30)	.31	2.15 (1.36,3.38)	<.01*	1.16 (0.74,1.82)	.52	2.05 (1.3,3.22)	<.01*	1.11 (0.71,1.75)	.64
Frequency of support												
*Continuous, vs null*	1.33 (0.85,2.08)	.21	1.44 (0.92,2.25)	.11	2.12 (1.37,3.31)	<.01*	1.06 (0.68,1.63)	.80	2.12 (1.37,3.30)	<.01*	1.15 (0.74,1.79)	.51
Psychological support												
*Yes, vs No*	1.15 (0.73,1.83)	.55	1.24 (0.78,1.98)	.36	1.69 (1.07,2.66)	.02*	1.02 (0.65,1.60)	.93	1.79 (1.14,2.81)	.01*	0.92 (0.59,1.45)	.73
Type of support												
*HCP vs none*	0.9 (0.47,1.74)	.75	0.97 (0.50,1.87)	.92	2.63 (1.23,5.61)	.01*	1.43 (0.74,2.78)	.28	2.50 (1.22,5.22)	.02*	0.99 (0.51,1.95)	.98
*Mobile vs none*	2.16 (1.09,4.31)	.03*	2.12 (1.06,4.23)	.03*	3.11 (1.47,6.55)	<.01*	1.46 (0.78,2.73)	.24	2.24 (1.54,3.25)	<.01*	1.49 (0.79,2.81)	.22
*Husband vs none*	1.51 (0.91,2.52)	.11	1.64 (0.98,2.75)	.06	1.99 (1.17,3.38)	.01*	1.93 (1.17,3.16)	.01*	1.93 (1.15,3.26)	.01*	1.20 (0.74,1.97)	.46
*All factors vs none*	2.15 (1.47,3.12)	<.01	2.34 (1.6,3.43)	<.01*	2.07 (1.42,3.01)	<.01*	1.52 (1.06,2.18)	.02*	3.82 (1.77,8.27)	<.01*	1.65 (1.15,2.36)	.01*

*Significant at 0.05.

ANC: antenatal care; CI: confidence interval; OR: odds ratio; PHC: primary healthcare center; Sig.: significance.

For self-care pre- and postdelivery, increased PHC visits, husband support for ANC visits, frequency of reminders, psychological support, and type of support all contributed to improved satisfaction. Specifically, increase in PHC visits led to a 64% increase in the odds of satisfaction pre- and postdelivery (OR = 1.64; CI = [1.15, 2.25]; p < 0.01). Although husband enrollment did not show significant effects, husband support for ANC visits significantly doubled the odds of satisfaction predelivery (OR = 2.15; CI = [1.36, 3.38]; p < 0.01) and postdelivery (OR = 2.05; CI = [1.3, 3.22]; p < 0.01). Additionally, the continuous reminders by the husband increased the likelihood of satisfaction with self-care both pre- and postpregnancy (OR = 2.12; CI = [1.37, 3.31]; p < 0.01). Psychological support also improved satisfaction, with a 69% increase predelivery (OR = 1.69; CI = [1.07,2.66]; p = 0.02) and a 79% increase postdelivery (OR = 1.79; CI = [1.14,2.81]; p = 0.03).

Regarding self and fetus care, all types of support were significantly associated with increased satisfaction pre- and postdelivery. Healthcare provider support was particularly impactful, increasing the odds of satisfaction with self-care predelivery by 2.63 times (OR = 2.63; CI = [1.23, 5.61]; p = 0.01) and postdelivery by 2.5 times (OR = 2.50; CI = [1.22, 5.22]; p = 0.02). Mobile support also played a key role, with women receiving mobile support being 3.11 times more likely to report satisfaction predelivery (OR = 3.11; CI = [1.47,6.55]; p < 0.01) and 2.24 times more likely postdelivery (OR = 2.24; CI = [1.54,3.25]; p = 0.02). Husband support further increased the odds of satisfaction with self and fetus care predelivery (OR = 1.99; CI = [1.17, 3.38]; p = 0.01) and postdelivery (OR = 1.93; CI = [1.15, 3.26]; p = 0.01). However, the combination of all factors significantly boosted satisfaction, with a two-fold likelihood of improvement in self and fetus care predelivery (OR = 2.07; CI = [1.42, 3.01]; p < 0.01) and 3.82 times greater likelihood of improvement in self-care postdelivery (OR = 3.82; CI = [1.77, 8.27]; p < 0.01).

Regarding satisfaction with baby health, an increase in PHC visits was significantly associated with a 35% increase in the odds of improvement (OR = 1.35; CI = [1.00, 1.38]; p = 0.05). Husband support (OR = 1.93; CI = [1.17, 3.16]; p = 0.01) and the combination of all factors (OR = 1.52; CI = [1.06, 2.18]; p = 0.02) were also positively linked with improved satisfaction with baby health.

Lastly, in correspondence to pregnant women with the service quality, increased PHC visits were associated with a 2.68 times higher likelihood of satisfaction (OR = 2.68; CI = [1.93, 3.71]; p = 0.01). Among the different types of support, the combined approach—including healthcare provider, mobile messages, and husband support—was particularly effective, yielding a 65% increase in the odds of satisfaction with service quality (OR = 1.65; CI = [1.15, 2.36]; p = 0.01).

## Discussion

The results indicated that the intervention led to high satisfaction levels among pregnant women across all assessment factors, with over 60% reporting improvement in all aspects, except for the satisfaction with baby's health postdelivery. The study further revealed that the enrollment of husbands in the intervention played a crucial role in enhancing his involvement, yielding higher levels of engagement. However, husband enrollment and engagement alone were not enough to improve the odds of satisfaction. Mobile digital prompts showed a high impact, while the combined approach involving multiple factors (husband, mobile, and healthcare provider) proved to be the most effective in enhancing satisfaction's odds.

### The influence of the engagement of the husbands on the satisfaction of the pregnant women

The results of this study underscore the pivotal role of husbands in providing essential support to their spouses during pregnancy. The data reveals that husbands who participated in the intervention were significantly *more* engaged in ANC support, actively reminding their wives about medical visits and offering consistent psychological assistance. These results suggest that low-cost digital health interventions can be instrumental in raising awareness and fostering greater involvement among husbands, ultimately creating a more supportive environment for pregnant women. Hazra et al. found that engaging the husband through biweekly digital prompts on maternal and child health (MCH) behaviors led to improved MCH behaviors, such as increased ANC and PNC checkups.^28^ By actively engaging partners, digital prompting not only enhances maternal wellbeing but also contributes to improved pregnancy experiences and outcomes. Musiimenta et al., suggested that leveraging mHealth to increase spousal involvement, through sending educational videos can enhance maternal health outcomes.^61^ Future programs should prioritize the inclusion of husbands in maternal care interventions, ensuring that they are equipped with the knowledge and resources needed to support their partners effectively.

### The influence of digital prompting on the satisfaction of the pregnant women

While the inclusion of husbands in digital health interventions has demonstrated promising outcomes, the findings of this study emphasize that achieving comprehensive maternal satisfaction requires a synergistic approach that integrates multiple support mechanisms. The results underscore the multifaceted nature of satisfaction among pregnant women, highlighting the critical roles of both digital prompting and a collaborative supportive environment, including husbands and HCPs. Similarly, mobile-based interventions—such as WhatsApp reminders and gestational age-specific weekly educational messages—were found to have a strong impact on multiple satisfaction factors, particularly in physical health, mental health, self- and fetus care, and self-care postdelivery. These interventions provide personalized, on-demand, and language-accessible support for the pregnant women, ensuring they receive the right information at the right time. This aligns with existing literature, which suggests that mHealth interventions can increase satisfaction with ANC and the number of ANC visits (4 or more ANC visits), improve the mental health and self-care of pregnant women and mothers, potentially reducing maternal morbidity and mortality.^17,62,63^ However, despite the effectiveness of digital prompting, the greatest improvements were observed when the support was at multiple levels (HCP, Mobile, Husband, and All factors), highlighting the value of a comprehensive, collaborative approach beyond digital solutions alone. Our findings are in alignment with the MatHealth App implemented in Uganda which demonstrated the effectiveness of digital prompts (appointment reminders), educational material, and HCP engagement in improving maternal health practices and increasing spousal involvement.^64^

### The influence of an integrated mHealth intervention on the satisfaction of pregnant women

The results of this study indicate that comprehensive, multidimensional support—including HCP engagement, mobile-based education, and spousal support—was the most effective strategy for enhancing satisfaction across all domains. This highlights the need for integrated intervention models that merge digital tools with interpersonal and institutional support structures to ensure that pregnant women receive holistic care. These insights advocate for a shift toward hybrid digital health models that combine mobile-based education with community-based engagement. While mHealth remains a powerful tool for maternal health, it is the intersection of digital interventions and human-centered care that maximizes impact. Future interventions should therefore focus on enhancing synergy between digital health solutions and collaborative, multistakeholder support systems to optimize maternal satisfaction and health outcomes.

### Limitations

This study faced a couple of limitations that may have influenced the findings. First, poor network connectivity and background distractions sometimes affected survey completion, as the survey was conducted via phone. These interruptions may have led to incomplete responses or reduced engagement. Second, targeted women were in some cases not the primary phone owners, but rather shared a phone with their husband or a relative. In the cases where the phone belonged to her husband or a relative, challenges were faced in reaching the intended participant directly. Moreover, some women declined to participate in the survey. These factors could have introduced response bias, as women who were harder to reach or who declined participation may have different experiences, perspectives, or levels of satisfaction compared to those who completed the survey, potentially affecting the generalizability of the results. While efforts were made to encourage participation, this nonresponse bias should be considered when interpreting the findings. In addition, exposures (husband support) and outcomes (satisfaction) were self-reported in the same phone call, hence, differential misclassification may bias the results.

## Conclusion

Digital prompting is a promising approach to improve disadvantaged women's satisfaction with their reproductive health journey. The effectiveness of these approaches is maximized when other key figures in the woman's reproductive health journey are involved. In particular, spousal support and the healthcare provider engagement have shown a great potential in improving the reproductive health journey of disadvantaged women targeted by digital prompting interventions aiming at enhancing their use of ANC services. Future interventions should prioritize this synergy to optimize maternal experiences and outcomes.

## Supplemental Material

sj-docx-1-dhj-10.1177_20552076251406650 - Supplemental material for Can digital prompting and the engagement of the husband influence the satisfaction of disadvantaged women with their reproductive health journey? A cross-sectional study from Lebanon Supplemental material, sj-docx-1-dhj-10.1177_20552076251406650 for Can digital prompting and the engagement of the husband influence the satisfaction of disadvantaged women with their reproductive health journey? A cross-sectional study from Lebanon by Shadi Saleh, Nadine Sabra, Nour El Arnaout, Asmaa El Dakdouki, Khaled El Iskandarani, Zahraa Chamseddine and Mohamad Alameddine in DIGITAL HEALTH
