# Supplementary material for: Can digital prompting and the engagement of the husband influence the satisfaction of disadvantaged women with their reproductive health journey? A cross-sectional study from Lebanon
Source: Digit Health. 2025 Dec 17;11:20552076251406650. doi: 10.1177/20552076251406650 (PMC13291574; doi:10.1177/20552076251406650)
Supplement: sj-docx-1-dhj-10.1177_20552076251406650 - Supplemental material for Can digital prompting and the engagement of the husband influence the satisfaction of disadvantaged women with their reproductive health journey? A cross-sectional study from Lebanon [file sj-docx-1-dhj-10.1177_20552076251406650.docx]

| Table S1. Demographics and Antenatal Care Utilization Patterns Among Pregnant Women assessed for satisfaction (n=683) | | | |
| --- | --- | --- | --- |
| Variables | | **n** | **%** |
| Age (mean, SD) | | 28.44(6.02) | |
| Gravida (mean, SD) | | 2.67(1.68) | |
| Residence Area (n, %) | |  |  |
|  | *Urban* | 194 | 28.40 |
|  | *Rural* | 489 | 71.60 |
| ANC Visits (n, %) | |  |  |
|  | *Less than 4* | 21 | 3.07 |
|  | *4 and more* | 662 | 96.93 |
| Main Encouragement for ANC visits (n, %) | |  |  |
|  | *No one* | 430 | 6.30 |
|  | *Healthcare worker/ provider (HCP)* | 49 | 7.17 |
|  | *Mobile Digital Prompt* | 212 | 31.04 |
|  | *Women’s husband* | 91 | 13.32 |
|  | *All factors Combined* | 288 | 42.17 |

| Table S2. Association of Sociodemographic Factors (Possible Confounders) with the Satisfaction Indicators (Improvement versus Non-Improvement) (n=683) | | | | | | | | | | | | |
| --- | --- | --- | --- | --- | --- | --- | --- | --- | --- | --- | --- | --- |
|  | **Physical Health** | | **Mental Health** | | **Self and Fetus Care** | | **Baby’s Health Post-Delivery** | | **Selfcare Post Pregnancy** | | **Service Quality** | |
| Demographics | ***N (%)*** | ***Sig.*** | ***N (%)*** | ***Sig.*** | ***N (%)*** | ***Sig.*** | ***N (%)*** | ***Sig.*** | ***N (%)*** | ***Sig.*** | ***N (%)*** | ***Sig.*** |
| Delta Age^¥^ Mean (SD) | -.35(.48) | 0.472 | -.25(.49) | 0.603 | .22(.49) | 0.64 | .47(.49) | 0.337 | .22(.49) | 0.641 | -.39(.46) | 0.399 |
| Delta Gravida^¥^ Mean (SD) | .03(.13) | 0.835 | -.01(.13) | 0.922 | .03(.137) | 0.814 | .08(.136) | 0.511 | .03(.13) | 0.81 | .09(.12) | 0.475 |
| Residence Area | |  |  |  |  |  |  |  |  |  |  |  |
| *Urban* | 126(65.0) | 0.784 | 129(66.5) | 0.804 | 139(71.7) | 0.174 | 97(50.0) | 0.127 | 137(30.0) | 0.195 | 119(61.3) | 0.242 |
| *Rural* | 323(66.1) |  | 330(67.5) |  | 324(66.3) |  | 213(43.6) |  | 329(70.0) |  | 276(56.4) |  |
| Health Facility |  |  |  |  |  |  |  |  |  |  |  |  |
| *PHC* | 344(66.9) | 0.215 | 349(67.9) | 0.440 | 343(66.7) | 0.346 | 235(45.7) | 0.651 | 336(73.9) | 0.160 | 298(58.0) | 0.911 |
| *Private/public clinic* | 103(61.7) |  | 108(64.7) |  | 118(70.7) |  | 73(43.7) |  | 119(26.2) |  | 96(57.5) |  |
| ANC Visits |  |  |  |  |  |  |  |  |  |  |  |  |
| *Less than 4* | 17(81) | 0.136 | 17(81) | 0.173 | 17(81) | 0.191 | 13(61.9) | 0.123 | 16(3.50) | 0.359 | 18(85.7) | 0.090 |
| *4 and more* | 432(65.26) |  | 442(66.8) |  | 446(67.3) |  | 297(44.9) |  | 441(96.5) |  | 377(57) |  |
| *¥.* Delta values in table represent differences between group shown and reference group not shown (example Improved vs Not Improved) | | | | | | | | | | | | |
